# Editorial: Secondary metabolites in beverage plant: metabolism, function, and regulation

**DOI:** 10.3389/fpls.2025.1762771

**Published:** 2026-01-06

**Authors:** Qunfeng Zhang, Ligui Xiong, Xuan Xu

**Affiliations:** 1 Tea Research Institute, Chinese Academy of Agricultural Sciences, Hangzhou, China; 2 National Research Center of Engineering Technology for Utilization of Functional Ingredients from Botanicals, Hunan Agricultural University, Hunan, Changsha, China; 3 Luxembourg Institute of Science and Technology, Hautcharage, Luxembourg

**Keywords:** beverage plant, chrysanthemum, *Clausena lansium*, coconut, metabolism, quality, secondary metabolites, tea

Plant secondary metabolites possess unique color, taste, and aroma characteristics that are well-aligned with consumer preferences for beverages. The accumulation of these compounds (e.g., flavonoids, terpenoids, and alkaloids) is a product of long-term evolution driven by plant acclimatization and natural selection (Liao et al., 2021). Consequently, secondary metabolites in beverage plants not only contribute distinctive sensory properties but also play crucial roles in plant development, such as acting as antioxidants for photoprotection, and endowing health-promoting effects (Ahmed et al., 2021). Nevertheless, the content and metabolic pathways of these secondary metabolites are highly sensitive to environmental conditions and are regulated by multiple factors, including plant variety, soil composition, growth environment, horticultural practices, harvesting time, processing techniques, and storage conditions (Yu et al., 2020). This complexity gives rise to diverse and intricate biosynthetic pathways of secondary metabolites in beverage plants (Wang et al., 2020). In conclusion, the study of secondary metabolites in beverage plants requires a comprehensive investigation into their metabolism, functional roles, and regulatory mechanisms.

This editorial seeks to provide a comprehensive overview of studies in this Research Topic addressing the metabolism, function, and regulation of secondary metabolites in beverage plants, with the aim of expanding our understanding of these compounds and their potential for enhancing flavor profiles and health benefits. The Research Topic comprises one review and eight original research articles investigating tea plants and their wild relatives, as well as multiple cultivars of coconut, wampee, and chrysanthemum. The nine articles systematically explore compound accumulation patterns, regulatory mechanisms, and biological functions, including studies on flavonoids, anthocyanins, gallic acid, strigolactones, selenium, and fluoride-related metabolites; investigations of developmental stages, plant age, genotypes, and exogenous factors; and identification of key genes, transcription factors, and metabolic pathways. These findings provide theoretical bases for molecular breeding, quality regulation, and functional product development in beverage crops. These studies adopt an integrative methodology combining metabolomics, transcriptomics, molecular techniques, and physiological validation.

For beverages, desirable flavor usually reflects abundant secondary metabolite content and appropriate combinations. Among these flavor compounds, flavonoids are major contributors and are conventionally classified into seven subclasses: flavonols, flavones, isoflavones, anthocyanins, flavanones, flavanols, and chalcones. In the metabolomic investigation conducted by Hou et al.*,* the flavonoid composition of coconut water from two distinct varieties was examined across six developmental stages. The study identified a total of 123 flavonoid metabolites, noting a general decline in total flavonoid content as development progressed. Notably, the Hainan local coconut variety exhibited significantly elevated flavonoid concentrations. Furthermore, 38 differential metabolites were implicated in flavonoid and secondary metabolite biosynthesis pathways, providing valuable insights for the breeding of coconut varieties with enhanced flavonoid content and the development of functional products. Chen et al. identified DEGs and differential metabolites linked to flower color of three “Boju” chrysanthemum varieties (pink-white, yellow-white, pure yellow) using transcriptomic and metabolomic analyses. Differential metabolites were primarily associated with isoflavonoid and flavonoid biosynthesis pathways.

The biosynthesis of secondary metabolites constitutes an integral component of the plant metabolic network, characterized by stringent and precise regulatory mechanisms. Due to substrate competition and functional interactions among various secondary metabolites, elucidating the interrelationships within this metabolic framework presents a considerable challenge. Wu et al. cloned and characterized *CsRAB*, an R2R3-MYB transcription factor from purple tea “Hongfei.” Its overexpression in *Arabidopsis* upregulates anthocyanin biosynthesis-related genes, leading to purple stems and higher anthocyanin contents. The study confirms *CsRAB*’s critical positive regulatory role in tea plant anthocyanin biosynthesis. Shi et al. reported that endogenous hormones regulate gallic acid biosynthesis in tea shoot buds/leaves across five developmental stages. Changes in D-erythrosyl-4-phosphate and shikimic acid contents affect gallic acid (GA) accumulation. Jasmonic acid, abscisic acid, auxin, cytokinin, and gibberellin inhibit GA biosynthesis via 23 hormone signal transduction factors and 16 transcription factors. Zhao et al. investigated anthocyanin and carotenoid biosynthesis in *Clausena lansium* peels using metabolomic and transcriptomic analyses by comparing brown-peel and purple-peel *Clausena lansium*. Purple peels showed higher carotene, total anthocyanins, and the delphinidin/cyanidin ratio, but lower chlorophylls. The upregulation of key genes promoted anthocyanin accumulation, while DFR substrate specificity explained the absence of pelargonidin. The findings elucidate molecular mechanisms underlying peel color transition from brown to purple. Shi et al. found that tree age influences black tea quality via metabolic regulation. Older trees exhibited higher flavonoid and catechin (especially EGCG derivatives) contents, lower specific amino acids (theanine, glutamate), and altered sugar/lipid levels. Transcriptomic analysis showed differential expression of phenylpropanoid pathway genes and transcription factors, aligning with distinctive flavor profiles of tea from older trees.

The accumulation of secondary metabolites is often essential for plant adaptation to environmental fluctuations. Consequently, factors such as environmental conditions, nutrient availability, and external stimuli can markedly influence both the concentration and metabolic pathways of secondary metabolites in plants, ultimately resulting in variations in product flavor profiles. Shen et al. found that exogenous strigolactones (SL) alleviate drought stress in tea plants by improving photosynthetic adaptation, mitigating cell membrane damage, and enhancing antioxidant responses via catechin metabolism regulation. *CsD27*, a key SL-biosynthetic gene negatively correlated with malondialdehyde (MDA) content, was identified; its overexpression in *Arabidopsis* reduced MDA content and increased the drought survival rate, revealing SLs’ dual role in stress tolerance and tea quality preservation. Li et al. investigated fluoride (F) accumulation in tea leaves of two cultivars. Cell walls were identified as the primary F storage site, with F predominantly bound to pectin’s amino and carboxyl groups and correlated with metal elements such as aluminum. This binding prevents F from entering cells, mitigating intracellular damage and serving as a key F tolerance mechanism. An et al. reviewed the role of selenium (Se) as an essential trace element in Se-enriched tea production. Se bioavailability in soil affects tea plants’ Se assimilation and transportation. Se regulates tea growth by modifying soil microbes, improving soil fertility, and enhancing tolerance to abiotic stresses. It also modulates secondary metabolism to shape Se-rich functional components, providing theoretical support for optimizing Se management in tea gardens.

We are confident that this volume will significantly advance beverage plant research by providing deeper insights into the mechanisms underlying the generation of the distinctive aroma and complex secondary metabolite composition of widely consumed beverages.
